# Related factors of renal injury in primary Sjögren's syndrome

**DOI:** 10.1186/s12979-023-00375-3

**Published:** 2023-09-21

**Authors:** Nan Duan, Zhiyan Li, Yong Fan, Yaping Jiang, Haixia Li

**Affiliations:** 1 Department of Clinical Laboratory, Peking University First Hospital, Beijing, 100034 China; 2 Department of Rheumatology and Clinical Immunology, Peking University First Hospital, Beijing, 100034 China

**Keywords:** Primary Sjögren’s syndrome, Autoimmune disorder, Renal injury

## Abstract

**Background:**

Primary Sjögren’s syndrome (pSS) is a common chronic systemic autoimmune disorder which primarily affects the exocrine glands. Patients may have extraglandular disease involving multiple organs, including the kidneys. This study aimed at investigating the clinical data and laboratory markers which were associated with renal function damage or renal involvement.

**Method:**

One thousand two hundred eighty-eight adult pSS patients from the Department of Rheumatology and Clinical Immunology were enrolled in this retrospective cohort study. And there were 334 patients of them followed up for more than two years for analyzing demographic, clinical data and laboratory markers. Statistical analysis was performed by R software (Version 3.6.2).

**Result:**

Nearly 95% of 1288 pSS patients were women, and the positive rates of anti-SSA (Sjögren's syndrome A) and anti-SSB were 63% and 27% respectively. 12% of the pSS patients presented renal involvement with eGFR < 60 mL/min/1.73 m^2^, and the mean age of hospital presentation, serum creatinine and urea were the highest (*P* < 0.001), and ANA (antinuclear antibody)-positive, anti-SSB-positive and anti-scl-70-positive were more prevalent in this group. Multivariate analyses showed that age, urea, chlorine and anti-SSA indicate a significant association with renal dysfunction. Potassium, sodium and Jo-1 were also confirmed to be related with decreased renal function. The receiver operating characteristic (ROC) analysis including the above factors showed a good performance on the evaluation of renal injury including eGFR < 60 mL/min/1.73 m^2^ and eGFR 60 -90 mL/min/1.73 m^2^ in pSS, with area under curve (AUC) values of 0.957 and 0.821, and high sensitivity (71.1% and 84.4%) and specificity (95.5% and 70.5%). After a more than two years follow-up of anti-SSA positive patients, 34.14% of them developed decreased renal function, and 13.58% of them experienced a progression of renal injury with a 23.64% decrease in eGFR.

**Conclusion:**

Age, urea, chlorine, and anti-SSA were highly associated with renal injury in pSS. Early screening for autoantibodies would be meaningful for evaluation and prevention of renal injury in pSS.

## Introduction

Primary Sjögren’s syndrome (pSS) is a common chronic systemic autoimmune disorder which primarily affects the exocrine glands, mainly the salivary and lacrimal glands [1]. The disease usually occurs among females (with a female-to-male ratio of 9:1) in their middle age with a peak incidence age of approximately 50 years old [2]. The vast majority of patients present with a combination of xerophthalmia and xerostomia caused by dysfunction of exocrine glands. However, besides exocrine glands involvement, pSS also presents with various extraglandular manifestations, including cryoglobulinemic purpura, non-erosive symmetrical arthritis, myalgia and fatigue. Even vital organs can be impacted, such as pericarditis, interstitial pneumonia, autoimmune hepatitis and primary biliary cirrhosis, polyneuropathy and eventually progressive renal disease, ranging from tubulointerstitial nephritis (TIN) to glomerulonephritis [1, 3]. TIN with tubular acidosis is the most common renal disorder, and membranoproliferative glomerulonephritis (MPGN) is the second most common renal manifestation in pSS [4].

Compared to other systemic complications, renal disease in pSS is less frequent and has been reported to occur in about 1% to 33% of the patients due to between-study differences in the population, nature of the study and criteria used for diagnosis [5–7]. In an observation of twenty-five patients with pSS who underwent renal biopsies, the diagnosis of SS preceded renal manifestations by a median duration of 5.5 years [8]. Renal involvement in pSS includes both tubular and glomerular injury, and is mostly characterized by TIN which occurs before or near the onset of sicca symptoms and evolves slowly as a result of epithelial disease with lymphocytic infiltration [9, 10]. The development of kidney damage in pSS is usually slowly and insidious, and may lead to delays in diagnosis and appropriate treatment.

Renal injury of pSS has long been known to be multifaceted and possibly underestimated. Although some associations have been described, the factors that predispose patients to the renal complication still remain unclear. It was reported that antibodies present before the onset of pSS symptom. Nearly 66% of patients were positive for ANA, rheumatoid factor (RF), anti-SSA and anti-SSB antibodies 4–6 years before symptom onset [11]. Clinical and morphological features of kidney complication in pSS have varied widely, and the relationship between renal injury and the autoantibodies or other associated factors is seldom investigated. Therefore, in the present study, we explored the demographic, immunological features and prevalence of renal injury in patients with pSS. Moreover, we investigated the clinical data and laboratory markers which were associated with kidney function damage to conduct a thorough disease analysis, evaluation and prevention for pSS.

## Materials and methods

### Subjects

**Fig. 1:**
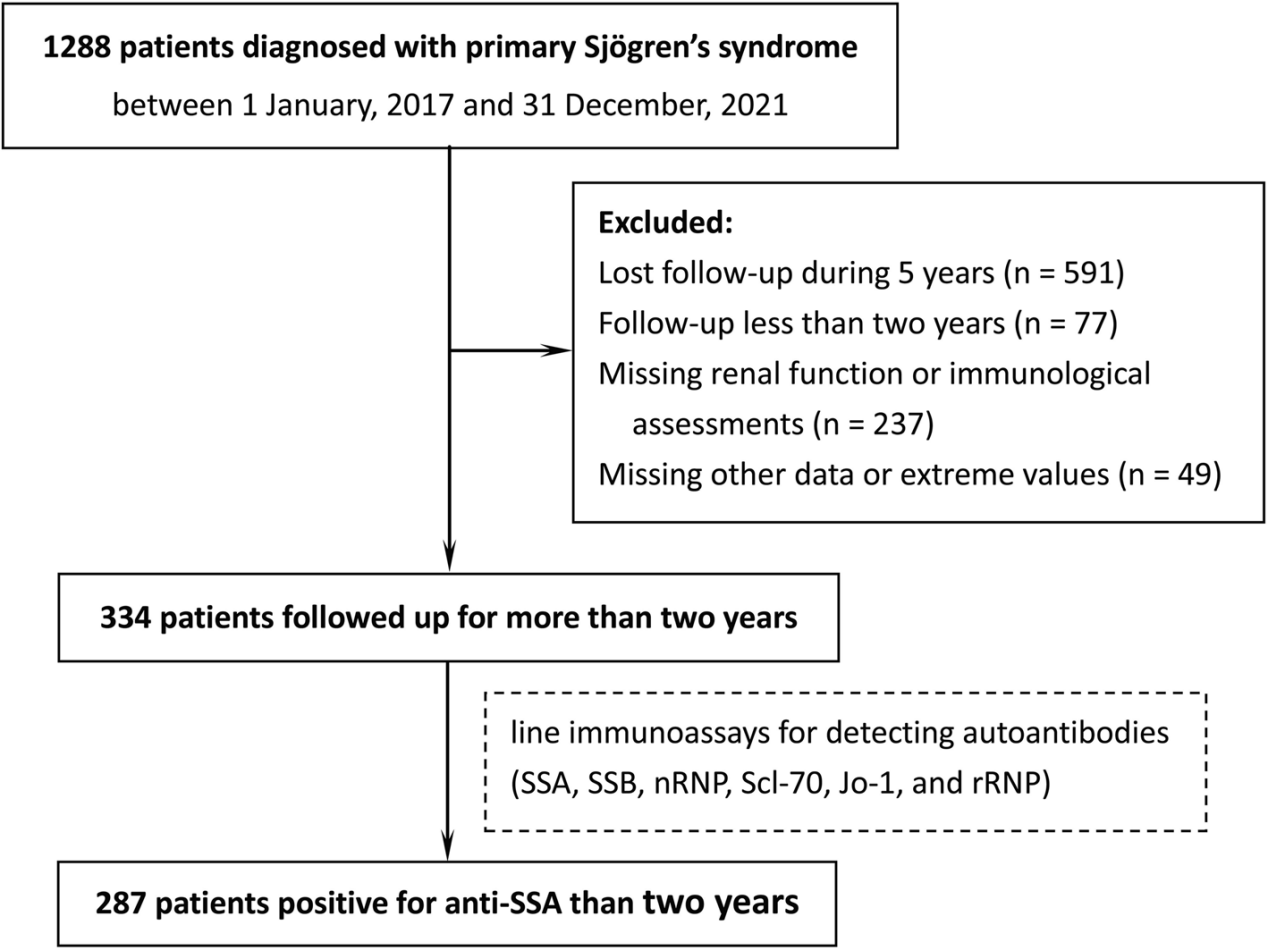
Flowchart of patient enrollment

We carried out a retrospective cohort study enrolled a total of 1288 adult patients satisfying the 2002 AECG (American-European Consensus Group) criteria [12] for diagnosis of pSS from the Department of Rheumatology and Clinical Immunology between 1 January, 2017 and 31 December, 2021 of Peking University First Hospital (a large-scale tertiary hospital). Demographic, clinical and laboratory data were collected from the pSS patients. Renal outcome was evaluated with serum creatinine levels and estimated glomerular filtration rate (eGFR) by the Chronic Kidney Disease Epidemiology Collaboration (CKD-EPI) equation [13]. In order to avoid potential interference from other secondary nephropathy, patients with suspected confounders of renal function such as diabetes mellitus, hypertension and malignancies were excluded in advance. Clinical characteristics and laboratory assessments of the study groups (totally 1288 patients) were summarized in Table 1 based on eGFR classes of the following eGFR strata: < 60, ≥ 60–90, and ≥ 90 mL/min/1.73 m^2^ as the G1, G2 and G3 group (all patients with eGFR < 60 mL/min/1.73 m^2^ were merged into one group). This eGFR value is considered as an evidence for defining a progressive and irreversible loss of renal function [14, 15]). In this study, 334 patients were followed up for more than two years with complete laboratory data from 1288 patients diagnosed with pSS (Fig. 1). A 20% or more decrease of eGFR was defined as progression of renal injury, and the enrolled subjects were then classified into progression group and non-progression group for comparisons. This study has been reviewed and approved by Institutional Ethics Committee of Peking University First Hospital (No. 2023-research-187).

**Table 1:** Clinical characteristics and laboratory assessments of the study groups

	**eGFR < 60**	**eGFR 60–90**	**eGFR ≥ 90**	***P* value**
	*N* = 159	*N* = 700	*N* = 429	
Age (years)	63.89 ± 12.94†	57.18 ± 11.95‡	43.12 ± 12.27	< 0.001
Female (n, %)	152, 95.6%	403, 93.9%	668, 95.4%	0.500
Creatinine (μmol/L)	127.75 ± 108.03†	76.68 ± 8.31‡	63.40 ± 7.98	< 0.001
Urea (mmol/L)	7.857 ± 3.643†	5.13 ± 1.35‡	4.39 ± 1.23	< 0.001
Potassium (mmol/L)	4.028 ± 0.575†	3.99 ± 0.35‡	3.88 ± 0.364	< 0.001
Sodium (mmol/L)	142.00 (140.00–144.00)†	141.56 (140.00–143.00)‡	140.14 (138.92–142.00)	< 0.001
Chlorine (mmol/L)	106.624 ± 3.855†	105.56 ± 2.27‡	105.01 ± 2.35	< 0.001
FPG (mmol/L)	5.29 (4.91–5.84)†	5.22 (4.87–5.58)‡	4.98 (4.71–5.34)	< 0.001
ANA-positive (n, %)	149 (93.7%)	640 (91.4%)	398 (92.8%)	0.584
	***N* = **114	***N* = **555	***N* = **356	
SSA-positive (n, %)	61 (53.5%)†	338 (60.9%) ‡	248 (69.7%)	0.002
SSB-positive (n, %)	34 (29.8%)	154 (27.7%)	88 (24.7%)	0.459
nRNP (n, %)				0.131
negative	109 (95.6%)	516 (93.1%)	322 (90.4%)	
positive	5 (4.4%)	38 (6.9%)	34 (9.6%)	
Scl-70 (n, %)				0.098
negative	110 (96.5%)	548 (98.7%)	353 (99.2%)	
positive	4 (3.5%)	7 (1.3%)	3 (0.8%)	
Jo-1 (n, %)		‡		0.002
negative	112 (98.2%)	554 (99.8%)	346 (97.2%)	
positive	2 (1.8%)	1 (0.2%)	10 (2.8%)	
rRNP (n, %)				0.602
negative	109 (97.3%)	536 (96.8%)	331 (95.7%)	
positive	3 (2.7%)	18 (3.2%)	15 (4.3%)	

Data are presented as mean ± standard deviation, median (25^th^ percentile-75^th^ percentile) or as n (%)

^†^ *P* < 0.05, eGFR < 60 compared to > 90

‡ *P* < 0.05, eGFR 60–90 compared to > 90

### Laboratory measurement

The systemic condition of study subjects was evaluated by a comprehensive review of medical history and a series of biochemical and immunological assessments. Blood samples were collected after an overnight fast of at least 12 h. Serum creatinine, potassium, sodium, chlorine, urea and fasting plasma glucose (FPG) were analyzed concomitantly on AU5800 automatic biochemical analyzer (Beckman Coulter, Inc., CA, USA). Serum creatinine levels were determined by Jaffe’s method. eGFR was calculated by the CKD-EPI equation by the Kidney Disease Improving Global Outcomes (eGFR_CKD-EPI_) [13]. Relevant immunological tests included antinuclear antibodies (ANA) using indirect immunofluorescence (IIF) on monkey liver tissue and Hep-2 cells (Euroimmun, Luebeck, Germany), and autoantibodies (SSA, SSB, nRNP, Scl-70, Jo-1 and rRNP) using line immunoassays by EUROLINE Plus (Euroimmun, Luebeck, Germany), etc.

### Statistical analysis

Statistical analysis was performed by R software (Version 3.6.2, https://www.r-project.org/) and the Deepwise & Beckman Coulter DxAI platform (https://dxonline.deepwise.com). Continuous variables were expressed as mean ± standard deviation or median (25^th^ percentile-75^th^ percentile), and One-way ANOVA or Kruskal–Wallis tests were used to compare the differences among G1, G2 and G3 group. Categorical variables were presented as frequencies with percentages, and the Chi-square (χ^2^) tests were applied to identify categorical variables among our study groups. Univariate and multivariate logistic regression models were created to analyze the relevant factors of renal function damage or renal injury in pSS. Odds ratios and 95% confidence intervals for risk were calculated on the basis of standard errors and model-variable coefficients. A nomogram of the model was generated based on the independent factors. Prediction models were evaluated using the receiver operating characteristic (ROC) curve. The area under the curve (AUC) was calculated, and an AUC of > 0.75 was considered to indicate good model performance. The *P* value of less than 0.05 was considered as indicating statistical significance.

## Results

### Clinical characteristics and laboratory results in pSS patients

One thousand two hundred eighty-eight diagnosed pSS patients from the Department of rheumatology and clinical immunology were enrolled in this study, and an overview of the baseline characteristics, biochemical and immunological assessments of included subjects was summarized in Table 1. 1025 patients had their first visit to our hospital performing autoantibodies testing (anti-SSA, anti-SSB, nRNP, Scl-70, Jo-1 and rRNP) by line immunoassays. Of the 1288 subjects included in the study, 1223 were women (95.0%), and 159 (12.34%) presented renal dysfunction with eGFR < 60 mL/min/1.73 m^2^. The main immunological markers of pSS, such as ANA, anti-SSA and anti-SSB were found positive in 92.16%, 63.12% and 26.93% of enrolled subjects, respectively. The mean age of hospital presentation (63.89 ± 12.94 years), serum creatinine (127.75 ± 108.03 μmol/L) and urea (7.857 ± 3.643 mmol/L) were the highest (all *P* < 0.001) and ANA-positive (ANA ≥ 1:100, 93.7%), anti-SSB-positive (29.8%) and anti-scl-70-positive (3.5%) were more prevalent in the G1 group (pSS patients with eGFR < 60 mL/min/1.73 m^2^) among all groups. Regarding the autoantibody profiles, there was no significant difference in the positive rate of ANA and anti-SSB among three groups, but anti-SSA-positive prevalence was much higher in those patients without renal damage (the G3 group: eGFR ≥ 90 mL/min/1.73 m^2^) compared to G1 group and G2 group (eGFR ≥ 60–90 mL/min/1.73 m^2^) (69.7% vs 53.5%, 69.7% vs 60.9%, *P* < 0.05).

### Relevant factors associated with renal injury in pSS patients

To confirm the possible related factors, the associations between the various factors in pSS patients with renal involvement were first analyzed by univariate logistic regression analyses. Since eGFR is calculated from serum creatinine, creatinine was not considered in the assessment. Besides age, urea, potassium, sodium, chlorine and FPG, the immunological index anti-SSA and Jo-1 were also significantly associated with renal function: there was a statistically significant association between anti-SSA and renal dysfunction in G1 group compared to G3 group, and anti-SSA, Jo-1 and decreased renal function in G2 group compared to G3 group. To avoid interference from potentially related factors, these factors associated with renal injury of pSS were further accessed using multivariate logistic regression analyses (Table 2). In the multivariate analysis, age, urea, chlorine and anti-SSA retained significant association with renal dysfunction in pSS. In addition, potassium, sodium and Jo-1 were also related with decreased renal function (the G2 group compared to G3 group).

**Table 2:** Multivariate analyses of associated factors in primary Sjögren’s syndrome

	**eGFR: ≤ 60 compared to > 90**		**eGFR: 60**–**90 compared to > 90**	
	**OR (95% CI)**	***P* value**	**OR (95% CI)**	***P* value**
Age	1.161 (1.110–1.214)	< 0.001	1.087 (1.070–1.105)	< 0.001
Urea (mmol/L)	2.612 (1.944–3.509)	< 0.001	1.337 (1.164–1.535)	< 0.001
Potassium (mmol/L)	0.54(0.223–1.306)	0.171	1.63 (1.019–2.610)	0.042
Sodium (mmol/L)	0.951 (0.796–1.135)	0.576	1.136 (1.042–1.239)	0.004
Chlorine (mmol/L)	1.33 (1.125–1.572)	0.001	0.958 (0.869–1.056)	0.385
FPG (mmol/L)	1.528 (0.941–2.480)	0.087	1.036 (0.840–1.278)	0.743
SSA	1.254 (0.520–2.562)	0.025	1.517 (1.040–2.214)	0.031
Jo-1	-	-	0.270 (0.080–0.584)	0.014

### Nomogram, AUC model and its performance

**Fig. 2:**
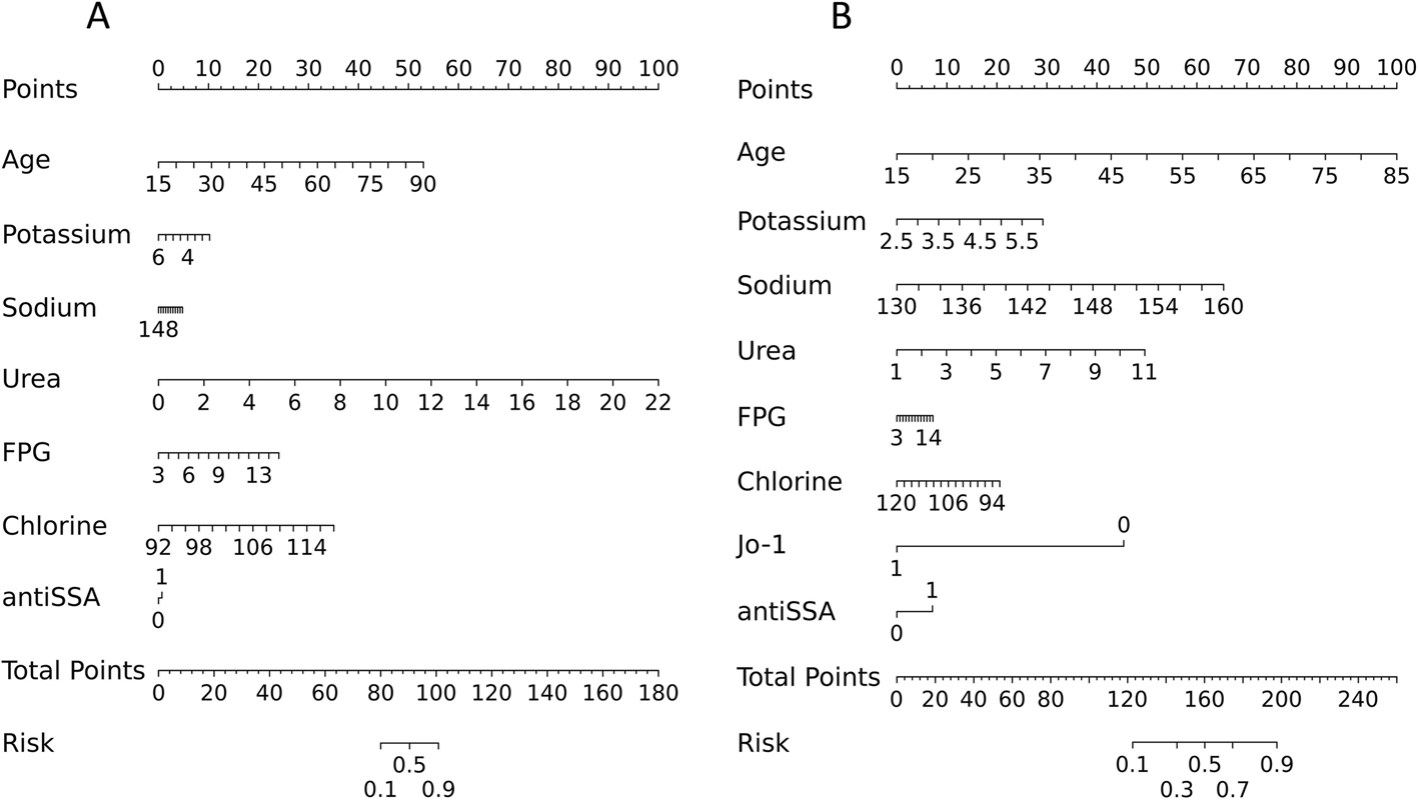
Nomogram graph of relevant factors of renal injury in primary Sjögren’s syndrome **A**. G1 group (pSS patients with eGFR < 60 mL/min/1.73 m^2^) compared to G3 group (pSS patients eGFR ≥ 90 mL/min/1.73 m^2^) **B**. G2 group (pSS patients eGFR ≥ 60–90 mL/min/1.73 m^2^) compared to G3 group

We included the above factors as the possible predictors of renal injury in pSS patients to build up a prediction model and presented a nomogram graph as shown in Fig. 2. Each level of every variable was assigned a score on the points scale, and the individual scores were summed to calculate a total score which reflected each pSS patient's probability of renal function damage.

**Fig. 3:**
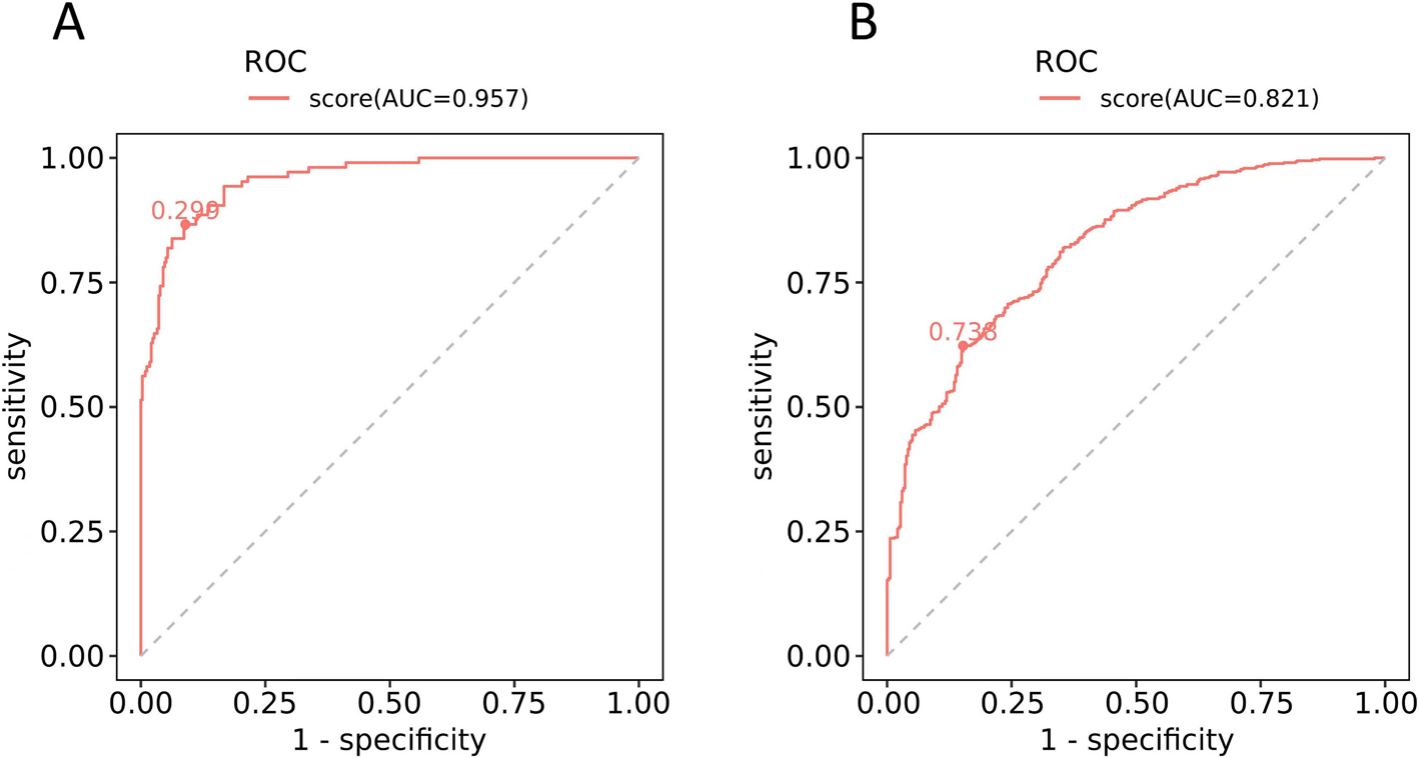
The receiver-operating characteristic curve of the multivariate logistic regression equation for evaluating renal injury in primary Sjögren’s syndrome **A**. G1 group compared to G3 group **B**. G2 group compared to G3 group

To confirm whether our mentioned factors could be used as valid predictors for renal injury in pSS, ROC analysis was applied. In the G1 group compared to G3 group, the AUC was 0.957, and the sensitivity and specificity were 77.1% and 95.5%, respectively (Fig. 3A). In the G2 group compared to G3 group, the AUC was 0.821 with a sensitivity of 84.4% and specificity of 70.5% (Fig. 3B).

### Progression of renal injury in anti-SSA positive patients

Among the 334 follow-up patients, 287 patients were positive for anti-SSA, and we selected biochemical and immunological data from this cohort before and after two years for comparative analysis (Table 3). After a more than two years follow-up, 98 (34.14%) subjects presented with decreased renal function and 39 (13.58%) of them experienced a progression of renal injury. In the renal injury progression group, eGFR was decreased about 23.64 (20.42–31.25)% and the levels of serum creatinine and urea were significantly higher than non-progression group (*P* < 0.01). Among patients with pSS, there was no significant difference in the positive rates of major immunological indicators including ANA, anti-SSB (51.28% *vs* 48.79%), nRNP (10.25% *vs* 11.69%), and rRNP (5.13% *vs* 7.66%) between the two groups. Moreover, in 7.69% (*n* = 3) of progression group and 6.05% (*n* = 15) of non-progression group, primary biliary cholangitis coexisted with pSS. The prevalence of autoimmune thyroid diseases in progression group (10.25%, *n* = 4) was lower than that in non-progression group (13.71%, *n* = 34) without statistically significant difference.

**Table 3:** Distribution of characteristics in anti-SSA positive subjects with more than two years follow-up (*N* = 287)

	**Renal injury**		***P* value**
	progression group (*N* = 39)	non-progression group (*N* = 248)	
Age (years)	54.00 (42.00–65.00)	53.00 (41.00–64.00)	0. 413
Female (n, %)	37, 94.87	232, 93.5	0. 510
Creatinine (μmol/ L)	79.50 (74.25–91.80)	68.95 (61.50–77.85)	< 0.001
eGFR change (%)	-23.64 (-31.25–-20.42)	4.71(-1.21–13.14)	< 0.001
Urea (mmol/L)	5.93 (4.80–7.49)	4.96 (4.07–5.96)	< 0.007
Potassium (mmol/L)	4.00 (3.68–4.31)	4.00 (3.74–4.25)	0. 942
Sodium (mmol/L)	141.20 (138.18–144.00)	141.10 (139.00–143.10)	0.723
Chlorine (mmol/L)	106.00 (104.00–108.00)	105.40 (104.00–107.80)	0.212
FPG (mmol/L)	5.25 (4.88–5.76)	5.14 (4.86–5.47)	0.137
ANA-positive (n, %)	39, 100	248, 100	-
SSB-positive (n, %)	20, 51.28	121, 48.79	0.772
nRNP-positive (n, %)	4, 10.25	29, 11.69	0.794
Scl-70-positive (n, %)	0, 0	1, 0.40	-
Jo-1-positive (n, %)	0,	0,	-
rRNP-positive (n, %)	2, 5.13	19, 7.66	0.815
Systemic lupus erythematosus	0	1, 0.4	-
Primary biliary cholangitis	3, 7.69	15, 6.05	0.694
Autoimmune thyroid diseases	4, 10.25	34, 13.71	0.554
Raynaud’s phenomenon	9, 23.08	41, 16.53	0.317

## Discussion

In this retrospective cohort study, we enrolled 1288 adult pSS patients from the Department of rheumatology and clinical immunology during five years (1 January, 2017 to 31 December, 2022). It was demonstrated that 95% of the pSS patients were women, and the positive rates of anti-SSA and anti-SSB were 63% and 27% respectively. 12% of the pSS patients presented renal injury with eGFR < 60 mL/min/1.73 m^2^. By multivariate analyses, age, urea, chlorine and anti-SSA indicate a significant association with renal injury in pSS. The ROC analysis included the above factors which showed a good performance on the evaluation of renal dysfunctiont and decreased renal function in pSS, with AUC values of 0.957 and 0.821, and high sensitivity and specificity. After a more than two years follow-up of anti-SSA positive patients, 13.58% of them experienced a progression of renal injury with a 23.64% decrease in eGFR.

As a systemic autoimmune disease, pSS is characterized by sicca syndrome secondary to infiltration of exocrine glands by lymphocytes [2, 10]. It usually occurs among middle-aged women. Patients may have extraglandular disease involving multiple organs, including the kidneys. pSS influences the kidney through lymphocytic infiltration of renal tubules or immune complex deposition, resulting in a series of clinical features [10]. Renal involvement is frequently misdiagnosed and presents a diagnostic challenge because of different factors, such as the insidious clinical symptoms or the condition that tubular dysfunction, which is the most prevalent alteration yet quite difficult to be recognized in pSS patients. Therefore, a particular focus on relevant factors or markers necessary to elicit kidney damage in pSS will facilitate an improvement in disease evaluation and prevention.

The present study showed that 95% of the pSS patients were women, as previous study documented that autoimmune diseases such as pSS, rheumatoid arthritis, and systemic lupus erythematosus occur more frequently in female than in male [16]. The positive rates of anti-SSA and anti-SSB, the main immunological markers of pSS, were 63% and 27% of enrolled subjects. This finding was similar to a French epidemiological study which reported prevalence rates of approximately 59% and 33% for anti-SSA and anti-SSB antibodies in pSS [17]. Although eGFR can be influenced by many factors including age, weight, medications and hydration status, it is still the best way to evaluate renal function, and renal outcome in most studies has been expressed by mean eGFR level [18]. In our cohort, 12% of the pSS patients presented renal dysfunction with eGFR < 60 mL/min/1.73 m^2^, which is consistent with a recent review showed that kidneys would be affected in about 5–14% of pSS cases according to several European studies [1]. However, some prospective studies found that kidney injury occurred in up to 27% of patients with pSS [19, 20]. This discrepancy in prevalence rates could be attributed to between-study differences in the diagnostic criteria, and also indicates that renal injury in pSS is an underdiagnosed problem. Various environmental and predisposing genetic factors may play an important role in an early onset of the disease which needs to be evaluated further.

In the G1 group (pSS patients with eGFR < 60 mL/min/1.73 m^2^), the mean age of hospital presentation, serum creatinine and urea were the highest among all groups. Several large cohort studies have reported that renal manifestations in pSS usually presented in patients aged ≥ 50 years [6, 20, 21], and it is suggested that yearly screening for urinalysis and serum creatinine is needed when manifestations of systemic disease are present [1, 22]. In our study, the G1 group had higher rates of ANA, anti-SSB and anti-scl-70 positivity though the difference failed to reach statistical significance probably because of the limited sample size. On the contrary, anti-SSA-positive prevalence was significantly higher in those patients without renal damage. Luo et al. from China also observed that patients with renal disease were found to have higher serum levels of anti-SSB, anti-scl-70, urea, creatinine and other molecules [23].

Renal involvement in pSS usually has an insidious progressive course but directly affects the prognosis of pSS patients. Thoroughly understanding the factors related to renal damage in pSS is necessary and may improve the management of patients. To further investigate the association between the various factors in pSS with renal injury, logistic regression analyses were conducted. Secondly, we incorporated these significant clinical factors into the establishment of a nomogram model using the logistic regression method. Our findings indicated that age, urea, chlorine and anti-SSA had significant association with renal dysfunction in pSS by univariate and multivariate analyses. Furthermore, potassium, sodium and Jo-1 were also confirmed to be related with decreased renal function. Recent researches also showed older age was a significant predictor of overt renal involvement [24], and other extraglandular manifestations in pSS, such as pulmonary or neurologic disease [25, 26]. Some factors were reported to be positively associated with kidney disease in pSS, including ANA, anti-SSA, anti-SSB, RF, low C3/C4, urea, creatinine, cystatin C, β2-microglobulin, etc. [1, 10, 27]. Among these factors, positive anti-SSA and/or anti-SSB antibodies are often considered immunological parameters of disease activity and extraglandular involvement in pSS. Previous researches and meta-analysis showed that the presence of anti-SSA antibody [28, 29] and anti-SSB antibody [30] were positively related to renal involvement, development or worse outcome of renal impairment in pSS. In our study, anti-SSA was observed to be positive associated with renal injury by multivariate analysis, which was also found in previous study showed antibodies anti-SSA and anti-SSB were associated with renal disease (particularly with TIN), and with poorer renal prognosis [6, 28], suggesting that pSS patients warrant careful workup for renal function. However, the presence of anti-SSA and RF positivity, low C3 levels are also reported as inversely associated factors [1]. Therefore, anti-SSA is either positive or inverse associations with the development of renal complications, and our founding supports a positive role of anti-SSA antibody presence in kidney disease progression. Many forms of kidney diseases, such as TIN, MPGN, membranous nephropathy, focal segmental glomerulosclerosis, IgA nephropathy, etc., have been reported to occur in pSS patients with biopsy-confirmed renal disease, with TIN accounting for approximately two-thirds of patients with pSS and kidney dysfunction [22]. In this study, a puncture biopsy was not performed after finding kidney injury and a further study will be needed by performing kidney biopsy to distinguish or exclude other causes of kidney injury. Notable clinical renal involvement in chronic TIN is often difficult to diagnose [22] and in some cases, renal biopsy is necessary for the definitive diagnosis of kidney involvement in pSS [1]. After analyzing the possible related factors of renal injury in pSS patients, we need further validations of its predictive effect. The ROC analyses were carried out in the same population. As a prediction tool, our model showed a good performance and discriminative ability on the evaluation of renal injury in pSS, with AUC values of 0.957 and 0.821, and high sensitivity of 71.1% and 84.4% respectively. This prediction model is easy and convenient to apply for renal injury prediction in pSS patients, as all factors included are easy to collect in clinical practice. Thus, we believe that the model can be used in daily clinical work to help doctors determine kidney function damage in pSS.

After a more than two years follow-up, 34.14% pSS patients with positive anti-SSA developed decreased renal function, and 13.58% of them experienced a progression of renal injury with an approximate 23.64% decrease in eGFR. Using eGFR events including eGFR decrease of 25% in the definition of kidney disease outcomes was appropriate to identify risk factors for CKD progression [31]. Another report showed that a reduction of 30% in eGFR was associated with a fivefold increased risk of end-stage renal disease, supporting consideration of lesser declines in eGFR (such as a 30% reduction over 2 years) as an alternative end point for CKD progression [32]. We analyzed the follow-up data and found that only 24 patients with pSS had a 25% or more decrease of eGFR over 2 years. Considering that the small sample size might affect the accuracy of the analysis and that the development of renal involvement in pSS is usually slowly and insidious, we chose a 20% or more decrease of eGFR over 2 years as a progression of renal injury. Among patients with pSS, the progression group had a higher positive rate of SSB and lower positive rates of nRNP and rRNP compared with non-progression group. In addition, the non-progression group had higher rates of autoimmune thyroid disease (ATD) though the difference failed to reach statistical significance, as patients with ATD were observed to have a lower prevalence of SSA and SSB antibodies but similar cumulative SS activity [33]. The prevalence of primary biliary cholangitis (PBC) was 7.69% and 6.05% in the progression group and non-progression group, which was consistent with previous reports that in approximately 3–9% of patients, PBC coexisted with pSS [3, 34].

A wide array of heterogeneous risk factors may contribute to chronic kidney disease (CKD) progression [14, 15]: 1) systemic/metabolic disorders including diabetes mellitus (DM), hypertension, connective tissue disease, autoimmune diseases, systemic sepsis, and gout, 2) kidney diseases such as glomerulonephropathy and tubulointerstitial nephritis, 3) cardiovascular diseases (CVD), and 4) obesity, as well as ageing of the population. CVD is one of the main causes of pSS death and increased risk of CVD is associated with a great variety of factors, such as age, gender, hypertension, DM, and so on [35]. A two-fold increased prevalence of DM was found in patients with pSS [36]. The enrolled patients have a significantly gradual increase in fasting plasma glucose as eGFR decreases, but the values are all below a normal level of fasting plasma glucose (Table 1), and thus effects of plasma glucose on the progressive loss of renal function can be excluded in the current study. Moreover, pSS patients with suspected confounders of renal function such as DM, hypertension and malignancies were excluded in advance to avoid potential interference. Hypertension is considered an independent risk factor for CVD in pSS patients and its prevalence is increased compared with the general population [37, 38]. In the present study, blood pressure values of the enrolled patients are below 140/90 mmHg, and a risk factor of hypertension for progression of renal injury can be excluded.

The strengths of our study comprise the use of data generated from a large-scale cohort, the availability of baseline medical information for participants, and our measurement are non-invasive and cost-effective. However, our research has certain limitations, partly because it was single-hospital design. Our study is also limited by retrospective observational design, and we could not differentiate patients with TIN from those with glomerulonephritis. Kidney biopsy is further needed to perform to differentiate TIN from glomerulonephritis, and distinguish or exclude other causes of kidney injury in the future study. Only 334 patients were followed up more than two years from 1288 adult pSS patients. Considering the small sample size of follow-up patient, participants may not be representative of the entire pSS population. Therefore, a multi-center, longitudinal, prospective study is required to clarify and confirm our findings.

In conclusion, age, urea, chlorine and anti-SSA were highly associated with renal injury in pSS. Early screening for autoantibodies would be needful for evaluation and prevention kidney function damage or renal involvement in pSS.

## Data Availability

The datasets used and/or analyzed in the current study are included in this article, further inquiries can be directed to the corresponding author.
